# Pain in Dairy Cattle: A Narrative Review of the Need for Pain Control, Industry Practices and Stakeholder Expectations, and Opportunities

**DOI:** 10.3390/ani15060877

**Published:** 2025-03-19

**Authors:** Steven Roche, Julia Saraceni, Laura Zehr, David Renaud

**Affiliations:** 1 ACER Consulting, Guelph, ON N1G 5L3, Canada; jsaraceni@acerconsult.ca (J.S.); lzehr@acerconsult.ca (L.Z.); renaudd@uoguelph.ca (D.R.); 2 Department of Population Medicine, Ontario Veterinary College, University of Guelph, Guelph, ON N1G 2W1, Canada

**Keywords:** pain, analgesia, anesthesia, cattle, bovine, ruminant

## Abstract

Pain is an adverse experience causing distress and decreased production in dairy cattle. Pain, and its associated distress, is also undesirable from an animal welfare standpoint. Consumers consider animal welfare an important issue; therefore, ensuring proper pain management, and by extension good welfare, is important to maintain the social acceptability of dairy production. However, while some painful procedures and diseases can be avoided, some are inevitable. When this is the case, it is important to provide pain mitigation where possible. Various dairy quality assurance programs provide guidance on pain management practices for producers; however, guidelines differ across countries and jurisdictions. This narrative review covers common painful conditions and procedures, including disbudding and dehorning, castration, calving and dystocia, surgeries, disease conditions, and lameness. Further, this paper reviews evidence of the necessity and efficacy of pain management in these cases, current uptake of pain management, and quality assurance program standards for addressing pain in dairy cattle. Overall, there are clear advantages to providing pain mitigation for some conditions and procedures. For others, gaps still exist in understanding the best methods for pain control. Further attention should be paid to understanding and reducing the barriers to adoption of pain management strategies, as it is crucial to minimizing pain in animals and ensuring productive and sustainable dairy production.

## Simple Summary

Painful conditions or procedures are often experienced by dairy cattle, whether because of management practices or disease. While prevention of painful procedures and diseases should always be a goal, there are still painful occurrences that are unavoidable. In these cases where pain cannot be avoided, pain control should be used to appropriately manage pain. Proper management of pain is important for animal health and welfare and is an important consideration for the sustainability of dairy production. This review discusses several of the common pain related practices in dairy production, as well as the recommended pain management practices, effects of pain management, industry program requirements, and current levels of pain management adoption by producers and veterinarians in Europe and North America.

## 1. Introduction

Pain is defined as an unpleasant sensory and emotional experience normally associated with tissue damage [1]. Dairy cattle frequently experience painful conditions, which include both management procedures (e.g., dehorning, castration, abdominal surgery) and disease-related pain (e.g., lameness, mastitis) [2]. While preventing pain is critical, many of these husbandry practices are needed, and diseases are unavoidable, leading to the necessity of pain control.

Although popular models of animal welfare vary (e.g., Five Freedoms, Five Domains, Quality of Life), they all act as frameworks to assess or evaluate the affective state or well-being of animals [3,4,5]. The Five Freedoms framework, originally developed by the Farm Animal Welfare Council in the United Kingdom, focuses on minimizing negative states by ensuring animals are free from hunger, discomfort, pain, distress, and have the freedom to express normal behavior [4]. In contrast, the Five Domains model expands upon this by incorporating both negative and positive experience across four domains (nutrition, environment, health, behavior) and one mental domain (affective states) [3]. Despite their differences, the various models and definitions of animal welfare circulating have reached consumers and become part of the discussion of food quality and value when it comes to animal products. Increasingly, consumers have concerns and expectations for the treatment and life of animals raised for food production, including a lack of disease and pain [6,7]. Additionally, the poor welfare of animals is a major reason why the public considers some animal systems unacceptable [8]. In this environment, pain management in dairy cattle is essential to ensure the sustainability of the dairy industry in the eyes of consumers.

Although in many parts of the world there are licensed anesthetic and analgesic products, such as nonsteroidal anti-inflammatory drugs (NSAIDs), there is limited use of these products to manage painful conditions [9]. Globally, the dairy industry has developed quality assurance (QA) programs to meet public expectations regarding animal welfare and farming practices. These programs vary in their scope and regulatory enforcement, with some being industry-led and voluntary, while others have mandatory compliance requirements. For example, the Farmers Assuring Responsible Management (FARM) in the United States of America is an industry-led, cooperative QA program, developed by the National Milk Producers Federation with input from veterinarians, animal scientists, and other industry stakeholders [10]. Similarly, proAction in Canada is also industry-led and informed by multiple stakeholders; however, compliance is mandatory for all dairy producers in Canada [11]. Another example is the Red Tractor in the United Kingdom, an independent QA scheme that includes specific requirements for pain management in common painful conditions that occur in dairy farming [12]. These programs have contributed to a substantial increase in the adoption of pain management in the dairy industry. Their pain mitigation standards are often informed by peer-reviewed literature, veterinary guidelines (e.g., American Association of Bovine Practitioners, Canadian Veterinary Medical Association), and regulatory frameworks (e.g., European Food Safety Authority reports). However, despite the progress, compliance and implementation remain inconsistent, with some regions and producers not fully adopting recommended pain management strategies.

The objective of this narrative review is to summarize the main painful practices and conditions that occur in the dairy industry and discuss the evidence on providing pain control, as well as the uptake of pain control methods, by producers and veterinarians. In addition, we will summarize the requirements of different quality assurance programs for dairy cattle and discuss mechanisms to motivate improvements in the use of pain control in dairy cattle.

## 2. Where Does Pain Occur in the Production of Cattle and Where Do We Start with Management?

In the production of cattle, some of the major pain-related practices include disbudding and dehorning, castration, calving and dystocia, surgeries, disease conditions, and lameness. These painful circumstances are difficult or impossible to completely eliminate, whether because of husbandry practices or nature. However, from an animal welfare standpoint, freedom from pain is a core standard of good welfare. Thus, it is important to mitigate the pain experienced from these unavoidable practices and conditions to ensure that cattle receive a life as free from pain as possible. Table 1 provides a summary of key painful conditions in dairy cattle, their recognized signs of pain, and recommended pain alleviation strategies. To further illustrate current pain management practices, Table 2 presents reported rates of local anesthesia and NSAID/systemic analgesia use among veterinarians and producers across various painful conditions. This comparison highlights gaps in pain management adoption, particularly among producers, and emphasizes the need for continued improvement in analgesic use. The conditions covered, including disbudding and dehorning, castration, lameness, parturition and dystocia, surgeries, and diseases, are explored in greater detail in the following sections. Each section presents evidence supporting pain mitigation strategies and provides insights into the uptake of pain management practices. It is important to note that not all listed pain relief methods are approved for every indication in all regions. Some NSAIDs and local anesthetics have specific regulatory approvals, while others are used in an extra-label manner depending on veterinary discretion and regional guidelines.

### 2.1. Disbudding and Dehorning

The removal of horns on cattle is a necessary practice that is performed to reduce the risk of injuries posed by horned cattle to both cattle and the humans that handle them [88,89]. Disbudding is performed in young calves before the horn bud is attached to the skull and typically involves thermal cautery (hot iron) or the application of caustic paste to destroy horn-producing cells [90]. In contrast, dehorning refers to the removal of the horn once it has grown to the point of attachment to the skull and often requires the use of gouging tools, saws, or wire, making it more invasive and painful than disbudding [90]. As outlined in Table 1, regardless of the method used, both disbudding and dehorning are painful. When no pain control is applied, clear behavioral changes are seen, such as an increased heart rate and decreased feed intake, along with neuroendocrine changes, such as increases in cortisol, which are indicators of acute pain [13,91]. With clear evidence of the pain associated with this painful condition, it is critical to ensure that the pain experienced when dehorning or disbudding is managed to ensure animal welfare is maximized.

The use of a local anesthetic block will eliminate the occurrence of acute pain in all methods of disbudding or dehorning [15,16,17]. The local anesthetic acts at the sodium channel to prevent the generation and propagation of nerve impulses or action potentials, thus preventing pain from occurring [92]. The typical local anesthetic used is lidocaine, which can provide pain relief within 5 min of administration and can last for up to 2 h [93]. However, there is a need for longer-term analgesia as the pain from disbudding and dehorning lasts for more than 2 h after the procedure is completed. Systemic analgesia provided through the use of non-steroidal anti-inflammatory drugs (NSAIDs) has been shown to reduce longer-term pain associated with disbudding or dehorning [16,17,18] These NSAIDs work by inhibiting the enzymes cyclooxygenase, lipoxygenase, and thromboxane that release mediators of inflammation in response to painful stimuli [93]. The combination of using both local anesthesia and NSAIDs provides short- and long-term pain relief. This is the best practice to mitigate pain for disbudding and dehorning [16] and is recommended by several veterinary organizations, such as the American Association of Bovine Practitioners [18] and the Canadian Veterinary Medical Association [90].

Several quality assurance programs responsible for overseeing animal care requirements for dairy farms require the use of pain control for disbudding and dehorning. Specifically, proAction, a mandatory national quality assurance program in Canada, requires that all producers administer a local anesthetic and an NSAID before all disbudding and dehorning practices since 2019 [94]. The Farmers Assuring Responsible Management (FARM) program in the United States of America also implemented new requirements as of July 2024, where all disbudding and dehorning practices must be performed with the use of pain control medication [10]. Although participation in the FARM program is voluntary, it is often required by milk processors and the majority of dairy farms in the USA are part of the FARM program. Outside of North America, other programs also require the use of pain management when disbudding or dehorning. For the Red Tractor dairy assurance scheme—the United Kingdom’s largest standard scheme—as of 2021, a combination of anesthetic and analgesic are required for almost all disbudding and dehorning procedures [12]. Clearly, with overwhelming evidence of the need for pain control, many quality assurance schemes are requiring its use.

As a result of these programs and the influence of external stakeholders, there has been an increase in the adoption of pain control for disbudding and dehorning (Table 2). Specifically, in a 2006 survey of American producers, only 12% and 2% of producers used local anesthetics and systemic analgesics, respectively [80], whereas a study from 2019 found that 21% of respondents used a local nerve block and 35% used an anti-inflammatory [81]. In Canada, in 2004, only 22% of producers reported using local anesthetics for dehorning or disbudding [82]; whereas, in a 2014 study, 62% and 24% of producers were using a local anesthetic and NSAID, respectively, when disbudding and dehorning their calves [83]. Among veterinarians, the use of pain control is substantially higher. In a 2014 survey of Ontario bovine veterinarians, 97% reported using local anesthetic and 48% used an NSAID when performing disbudding or dehorning [84]. Similar trends were observed in the UK, where 98% of veterinarians provided local anesthetic and 56% administered an NSAID when disbudding calves. Further supporting this trend, a survey of 569 veterinarians in the United States found that 65–77% consistently used local analgesia for dehorning, with usage increasing as cattle age. However, the use of systemic analgesia was lower, ranging from 39% in calves under two months to 56% in older cattle [95]. Despite these improvements, there remains room for further adoption of pain management. With increasing pressure from quality assurance programs and external stakeholders, continued uptake of effective pain control strategies is expected.

Breeding polled cattle may be an alternative where disbudding and dehorning are not needed. This has been commonly recommended as a measure to reduce the need to use these painful procedures and has become common in beef cattle production [96]. However, uptake has been poor, with few American and Canadian dairy producers using polled genetics [84,97]. This is likely due to producers’ concerns regarding the genetic quality of polled sires and the lack of availability of polled genetics [84]. As improved genetics become available in polled sires, there could be an increased North American uptake.

### 2.2. Supernumerary Teat Removal

The presence of supernumerary teats is a common congenital anomaly in cattle [19]. These extra teats can be problematic if they are located near a functional teat, as they may interfere with milking and increase the risk of mastitis [20]. Although limited research has evaluated the pain associated with supernumerary teat removal, this procedure is perceived as painful, and pain management is recommended through the administration of a local anesthetic at the base of the teat [17,21]. Despite this, pain control is not widely used. In New Zealand, only 57.1% of veterinarians believed that local anesthesia should be provided for most calves undergoing supernumerary teat removal, and just 44.3% thought systemic analgesia was necessary [21]. Similarly, in Germany, 90% of farms removed supernumerary teats without local anesthesia [22], and a 2007 survey of Canadian farmers found that only 6.8% of farms used local anesthetics for this procedure [23]. The low uptake of pain management may be due to the absence of explicit pain control requirements for supernumerary teat removal in some quality assurance programs [10,11].

### 2.3. Castration

Castration, while not traditionally performed on dairy farms, is increasingly used for non-replacement calves, those not needed for milk production, and those raised for beef [24]. Like disbudding, it is a painful condition that causes acute and long-term pain, particularly when performed without pain relief (Table 1) [25]. Rubber ring castration results in prolonged pain, with affected calves showing reduced weight gain, lower starter intake, less time lying down, and more frequent licking of lesions compared with surgical castration [26]. While local anesthesia can effectively control the acute pain, chronic pain may persist for weeks following rubber ring castration [85]. Due to animal welfare concerns, rubber ring castration is subject to varying regulations worldwide. For example, in the United Kingdom, it is restricted to calves under seven days old [85], whereas in Lithuania, it is banned due to concerns about its humaneness [85]. Lidocaine-impregnated bands are a recent innovation that may provide sustained pain relief. Studies have shown that these bands maintain effective tissue concentrations of lidocaine for at least 28 days, compared to injectable lidocaine, which only offers short-term anesthesia for up to 60 min [98,99]. However, further research is needed to assess their effectiveness in reducing pain-related behaviors, especially when combined with NSAIDs, as well as to evaluate withdrawal times associated with their application.

Multimodal pain relief through the use of local anesthesia and an NSAID is likely best at achieving proper pain management. Stafford et al. [100] used a combination of local anesthesia and ketoprofen, an NSAID, and found that this combination eliminated the cortisol response to castration. Further, Martin et al. [28] found that combining a local anesthetic with an NSAID not only reduced cortisol but also pain-relative behaviors, such as hunched standing, and improved calf gait. However, other studies, such as Webster et al. [29], have found no difference in cortisol and other behavioral markers of pain when multimodal pain management was used. Hence, additional studies are needed to determine the best management practice for pain relief when castration is completed, especially when rubber ring castration is used due to the long-lasting pain associated with the technique.

In North America, dairy quality assurance programs have implemented standards related to castration that include the use of pain control for this procedure. Both the FARM Program and proAction, the national dairy quality assurance programs for the U.S. and Canada, respectively, outline specific program requirements for animal health practices, including castration. Version 5.0 of FARM Animal Care requires that pain mitigation is provided for castration and that the farm’s written herd health plan includes an effective protocol for castration if performed on farm [10]. Similarly, the proAction program outlines that a documented standard operating procedure (SOP) for castration must be established and must include appropriate pain control [11]. The proAction program specifies that the use of sedation on its own does not constitute appropriate pain control. Both programs recommend that castration be performed as early as possible.

Despite evidence that there is pain associated with the castration of young calves, approximately 25% of UK cattle practitioners provided NSAIDs to >50% of calves that were castrated [9]. Similarly, in a survey of veterinarians and producers in the United States [84], only 32–48% of veterinarians consistently used local analgesia for surgical castration, depending on calf age, while 29–42% used systemic analgesia. In contrast, producer uptake was significantly lower, with only 10–13% using local analgesia and 8–10% using systemic analgesia across all age groups. Hence, it is clear that an increased uptake in the use of pain management by both veterinarians and producers when conducting this procedure would improve animal welfare.

### 2.4. Lameness

Lameness is a common condition that impacts dairy cattle, with an estimated prevalence of 13 to 55% of dairy cattle being lame on dairy farms worldwide [30,101,102]. There are many behavioral signs associated with lameness that indicate the pain that occurs with this condition (Table 1). Specifically, lame cows will arch their back, hang or bob their head during locomotion, shorten or lengthen their stride, and change their willingness to walk [30]. In addition, cattle will redistribute their weight away from a limb with discomfort as an adaptation to pain [31]. There are also physiological changes that occur following lameness, including an increased heart rate and elevated level of cortisol [32]. Many of the lesions that are responsible for causing lameness are located in hooves [33,34], which is the reason for focusing on claw lesions in this review.

Early identification and treatment of the underlying cause of lameness is critical for the management of pain and to improve recovery [35]. Therapeutic trimming consists of removing all necrotic and loose or undermined horn to create an aerobic environment and reduce the chance of having an abscess form. Adjusting weight bearing, where a block is applied to a healthy claw, will provide relief for the injured claw and reduce pain for the cow while they are recovering [36,37]. Therapeutic trimming of cows identified with lameness is critical to achieving a high rate of recovery from lameness and reducing or stopping pain from the condition; however, recovery is dependent on the severity of the lameness, with severely lame cows being less likely to recover [38]. In addition to corrective trimming and hoof blocks, the use of analgesics has also been explored as a mechanism to control pain; however, few trials have been conducted, especially with respect to providing NSAIDs at the time of diagnosis [39]. Of the trials that have been completed, improvements have been demonstrated. Specifically, following an injection with the NSAID ketoprofen in lame cows, Flower et al. [87] noted more even weight distribution in all four limbs and a mild improvement in lameness score, whereas Chapinal et al. [103] found reduced variation in weight distribution. In addition, Thomas et al. [104] found that the combination of a corrective trim, hoof block, and injection with ketoprofen improved lameness resolution 35 days following treatment compared to just a corrective trim. To provide further evidence for using NSAIDs as lameness treatment, Wilson et al. [40] found that providing lame cows with a 3-day course of ketoprofen, in combination with a therapeutic trim and a hoof block on the sound claw if needed, led to a lower risk of being culled compared to lame cows that received a therapeutic trim and hoof block if needed. Benefits have also been shown in the treatment of acute cases of digital dermatitis, where lame cows receiving ketoprofen at diagnosis with digital dermatitis in combination with topical oxytetracycline had increased milk production and lower odds of remaining lame compared to cows that just received topical oxytetracycline [41]. Cumulatively, from the research completed to date, providing appropriate therapy of the condition in combination with an NSAID may alleviate the pain experienced with this painful condition and improve recovery.

On-farm assessments are requirements of both the FARM and proAction quality assurance programs. Locomotion, or mobility scores, are required as part of these on-farm assessments, where animals are evaluated and scored to determine the prevalence of lameness on the farm. Corrective actions are required for farms that fall below the acceptable threshold for lameness prevalence. For the proAction program, 90% or more of observed animals must be sound in order to avoid a corrective action [11]. Similarly, the FARM Program requires that no more than 5% of the lactating cows observed a score as severely lame and no more than 15% score as moderately lame on the FARM locomotion scorecard [10].

With respect to pain control surrounding the treatment of lameness, there seems to be a higher proportion of veterinarians and producers using some form of pain management compared to many of the other painful conditions reviewed. In the UK, approximately 60% of veterinarians used an NSAID treatment in more than 50% of sole ulcers that they treated [9], whereas in a U.S. survey of veterinarians and producers, only 9% never used systemic analgesia, with 24%, 12%, 27%, and 22% sometimes, half of the time, most of the time, or always using systemic analgesia, respectively [84]. In Ireland, on pasture based dairy farms, 80% of producers would like a cow to receive an NSAID when a sole ulcer is diagnosed, whereas 75% of veterinarians used an NSAID in >50% of sole ulcer cases [42]. From the available information, it seems that pain control is provided more commonly to cows with lameness than with other common painful conditions (Table 2). This is likely because the clear physical manifestations of lameness, such as limping, reluctance to move, and abnormal weight distribution [30], make it easier to recognize and treat as compared to other conditions where signs of pain may be more subtle.

### 2.5. Parturition and Dystocia

Parturition is a necessary event that occurs in dairy cattle to lead to the onset of lactation. However, there is limited research into the welfare impacts of this event, although most veterinarians and producers feel that this is a painful and stressful event [9]. Most research surrounding pain at parturition in the dairy sector has focused on dystocia, defined as a difficult or abnormal calving. Reports of dystocia prevalence in dairy cattle range from 2 to 7% [105]; however, the level of calving assistance is much higher than this, with up to 50% of calvings reportedly requiring assistance [106]. Some studies have highlighted the occurrence of pain as a result of dystocia, mostly through observation of specific behavioral changes (Table 1). Specifically, cows with difficult calvings consume less feed and transition from standing to lying positions more frequently than cows without dystocia [43]. Others have also found that assisted cows took longer to stand after parturition and spent less time self-grooming postpartum when compared to cows that were not assisted [44,45]. Although these behavioral changes are subtle, many of these changes are indicators of pain [46,47].

The use of NSAIDs have shown promise In mitigating some of the effects of dystocia, or pain associated with parturition in general. In a study that administered either no treatment or ketoprofen to cows within 3 h following parturition, cows treated with ketoprofen spent less time in lateral recumbency and more time with their head rested when in sternal recumbency, which is a behavior associated with comfortable resting [48]. Another study found that post-calving administration of meloxicam to eutocic first lactation animals led to an increase in activity for the first 2 d post-calving when compared to eutocic primiparous controls [48]. Similarly, Barragan et al. [50] found that acetylsalicylic acid administration to eutocic cows increased activity post-calving compared to untreated eutocic controls. However, the regulatory status of acetylsalicylic acid varies by region, with no current approval from the Food and Drug Administration for use in cattle in the United States [51]. Newby et al. [107] also found behavioral changes when cows were administered meloxicam following a dystocia, where assisted cows treated with meloxicam visited the feed bunk more often and spent more time feeding than untreated controls. With respect to production parameters, Swartz et al. [108] found that the administration of meloxicam before calving led to a higher amount of milk fat, protein, and lactose than an untreated control. In addition, cows that did not need assistance but had received meloxicam prior to calving produced more milk (+6.8 kg/d) than controls for the first 15 weeks of lactation. Other studies found similar results, with Carpenter et al. [109] showing meloxicam administration after calving to lead to an increase in daily milk yield of 4 kg/d, whereas administration of sodium salicylate to mature cows led to cows producing 3.5 to 8 kg/d more milk, as well as more milk fat and protein during a 305-day lactation [54,110]. In addition, Shock et al. [54] found that cows that had been administered meloxicam produced 0.64 kg/d more milk over the first 3 DHI test days, had a lower risk of subclinical mastitis, and were less likely to be culled or die in early lactation compared to untreated controls. It is likely that these effects resulted from the inhibition of calving-mediated inflammation, as an elevation of inflammatory mediators in mice has been shown to increase apoptosis of milk-producing epithelium [50]. No studies have found a benefit to administering NSAIDs around parturition in terms of reducing the frequency of clinical disease [49,107,108,109,110]; however, the administration of flunixin meglumine around the time of calving has been shown to increase the risk for retained placenta, metritis, and stillbirths [111]. In addition, pre-calving use of acetylsalicylic acid led to a higher incidence of retained placenta when compared to untreated controls [55]. As both flunixin and acetylsalicylic acid have greater affinity for COX-1 inhibitors, this is likely the reason for a higher level of adverse effects. Meloxicam, which is a preferential COX-2 inhibitor, has been found to have no adverse effects regardless of the timing of administration [53].

Dystocia is recognized as a significant health event by the FARM Program, with a standard requiring that each farm’s herd health plan includes an effective protocol for difficult calvings [10]. The protocol is expected to include details, such as when to intervene and the appropriate equipment to use when assisting an animal that is experiencing dystocia.

Similar to the provision of pain control for cases of lameness, producers and veterinarians also commonly provide pain control in cases of dystocia (Table 2). Specifically, for UK veterinarians, approximately 60% will provide an NSAID in >50% of dystocia cases [9], whereas 73% and 68% of Irish producers and veterinarians would like to or do provide an NSAID to >50% of cases of dystocia, respectively [42]. It is unclear whether veterinarians or producers provide pain control in other parts of the world for dystocia; however, given the painful nature of the condition, future research should try to ascertain how commonly pain control is used outside of the UK and Ireland for dystocia.

### 2.6. Abdominal Surgeries

Abdominal surgical procedures, including caesarean sections and right flank laparotomies, can be performed on both an elective and emergency basis, leading to variable degrees of pain and distress (Table 1). The best way to improve welfare in surgical cases is to prevent pain from occurring [112]. During surgery, acute pain is due to tissue damage that results from cutting and manipulating tissue. Following surgery, inflammation will occur as a result of the tissue damage, causing additional pain. Hence, multimodal pain management, where a combination of drugs with different pharmacologic classes is used, is best to prevent acute pain and post-operative pain. Local anesthetics are the most common analgesic drug used for surgery, where they desensitize tissue to prevent acute pain from occurring [56]. Non-steroidal anti-inflammatory drugs are also commonly used; however, there are few clinical trials that have been conducted to show the benefit of using them. Of the trials that have been conducted, most find a benefit. Following a caesarean section, cows that received meloxicam were shown to spend more time lying down after surgery, suggesting that they may have increased comfort [57]. However, the use of flunixin meglumine following a c-section in cattle can increase the risk of a retained placenta [58]. Beyond c-sections, there is evidence that the use of an NSAID can be useful to manage pain in other abdominal surgeries. Specifically, Newby et al. [59] found that cows that received ketoprofen following left displaced abomasum surgery were more likely to begin consuming fresh feed during the first 3 days following surgery. However, this effect was reported by producers and feed intake was not directly measured, warranting further investigation. A similar study was conducted by Newby et al. [60], where ketoprofen was given at rumen fistulation surgery and again 24 h later; cows that received ketoprofen were more likely to lie on the left side where the surgical site was compared to the control cows. In addition, cows receiving ketoprofen had less tail flicking behavior, which is an indicator of pain. Finally, when using intravenous ketoprofen on the day of surgery followed by oral ketoprofen for 4 days following surgery, cows that received the NSAID had higher heart rate variability compared to the control group. This indicates that animals in the control group had higher sympathetic activity, likely due to untreated post-operative pain [61]. From the research highlighted above, there is evidence that sustained use of NSAIDs can help mitigate pain resulting from surgery. It is important to note, however, that even despite NSAID use, signs of pain can still be noted [59]. Therefore, additional research is needed to further our understanding of how to better manage postoperative pain in dairy cattle.

As part of each participating farm’s herd health plan (HHP), the FARM Program requires a written protocol that outlines standard operating procedures around the treatment of common diseases, such as displaced abomasum [10]. It is a requirement of the program that the HHP is reviewed and signed annually by the farm’s veterinarian of record.

The majority of producers and veterinarians use pain control for abdominal surgeries, likely because the level of perceived pain is high (Table 2) [9]. In veterinarians in the UK, approximately 80% and 70% used an NSAID in more than 50% of caesarean sections and LDA surgeries, respectively [9]. In Irish producers, 98% and 90% would like a cow to receive an NSAID for a caesarean section and LDA surgery, respectively; whereas, for veterinarians, 77% and 72% would use NSAIDs in >50% of caesarean sections and LDA surgeries [42]. In a survey of veterinarians and producers in the United States of America, 89% respondents always used local anesthesia for abdominal surgery and 61% always used an NSAID [84].

### 2.7. Disease

Despite how common disease occurs in dairy cattle, there is limited research on the impact that disease has on pain in cattle. There is, however, some work that has been conducted surrounding mastitis, metritis, and calf diarrhea. Mastitis is the most common disease occurring in dairy cattle, with a reported incidence of 24.4 cases of clinical mastitis found per 100 cow-lactations on 37 large dairy farms in Wisconsin, USA, more than twice the incidence rate to the next most common disease [62]. It is also documented to be painful (Table 1), with affected cows having higher heart and respiratory rates, higher levels of cortisol, larger hock to hock distances, and increased sensitivity to mechanical pressure stimulus on the leg closest to the affected quarter compared to cows without mastitis [63,64,65]. Although it is clear that the condition of mastitis is painful, minimal research has been completed to understand the best pain management practices. In LPS endotoxin challenge models, the use of flunixin meglumine has been shown to reduce signs of depression, decrease rectal temperature, increase rumen motility, and decrease heart rate [66,67,113,114]. Similarly, meloxicam administration following LPS challenge reduced udder edema, rectal temperature, and pain sensitivity while increasing rumen contractions [67,114]. In naturally occurring cases, fewer trials have been conducted. In mild cases of mastitis, meloxicam therapy with an antimicrobial was found to reduce culling and somatic cell count while improving cure rates and reproductive performance compared to solely an antimicrobial [68,69]. When ketoprofen is used for five consecutive days following a case of mastitis, cows returned to 75% of their daily milk production, had an improved recovery rate, and were less likely to be culled compared to an untreated control [70]. Based on the available evidence, NSAID use in cases of mastitis have been shown to have substantial positive effects on not only the welfare of the cows but also their productivity [71]. Despite this, when it comes to the use of NSAIDs for cases of mastitis, lower overall use has been reported than for other painful conditions (Table 2). In Ireland, only 26% of producers would like a cow to receive an NSAID for mastitis and just 42% of veterinarians use an NSAID in >50% of cases of mastitis [42]. Similarly, in the United States, only 13% always used NSAIDs in each case of mastitis and only 22% used it most of the time [84]. Based on this, there may be opportunities to encourage the use of NSAIDs in cases of mastitis.

Metritis is a painful inflammatory condition of the uterus, affecting 10 to 30% of cows within the first 21 days after calving [72]. It is characterized as an enlarged uterus with the presence of watery, fetid, red–brown vaginal discharge, with or without a fever [73]. Although metritis is not always recognized as a painful condition, evidence suggest that affected cows exhibit physiological and behavioral indicators of pain (Table 1). Specifically, palpation of the uterus in cows with metritis elicits a greater back arch and increased heart rate, both indicative of discomfort and pain [115]. Overall, there is a scarcity of evidence for the use of NSAIDs to control pain associated with metritis. Lomb et al. [74] used meloxicam in addition to an antimicrobial at the onset of metritis and found that meloxicam-treated cows had a greater number of visits to the feed bunk but less dry matter intake in the first 24 h following treatment compared to cows that solely received antimicrobials; however, from day 1 to day 5 following treatment with meloxicam, there was an increase in the number of meals compared to the placebo cows. Amiridis et al. [75] also found improvements in the clinical condition when flunixin meglumine was given at the onset of metritis, where a reduction in fever, faster resolution of the clinical condition, improved uterine involution, and calving-to-first-detected-estrus was found compared to untreated controls. Others have found minor differences when flunixin meglumine was used in combination with antimicrobials, such as a reduction in haptoglobin [76], or no differences [77] compared to solely an antimicrobial. Pohl et al. [78] compared solely using ketoprofen to an antimicrobial and found that ketoprofen-treated cows were more likely to require an extended treatment for metritis; however, overall, antimicrobial use was lower, and no differences were noted in the incidence of purulent vaginal discharge, milk yield, or reproductive performance. Similarly, Paiano et al. [79] reported no differences in health, milk yield, or reproductive performance when using ketoprofen instead of antimicrobials, supporting the potential use of NSAIDs as an alternative to antimicrobials, suggesting that NSAIDs could be used instead of antimicrobials. Based on the available evidence, future research should continue to evaluate the utility of NSAIDs to reduce pain associated with metritis and understand how commonly they are used by producers and veterinarians.

Diarrhea and respiratory disease are common health challenges in young calves. Across 104 dairy farms in the United States [116] and 147 farms in Canada [117], approximately 30% of calves receive treatment for these diseases. Despite how frequently they are treated for these conditions, little research has been conducted to determine whether these are painful conditions. It is clear that there are behavioral changes—such as reduction in milk consumption, drinking speed [118] and lying behavior [119]—that precede disease; however, these changes have not been tied to measures of pain. In addition, few papers have evaluated the effect of an NSAID as a supportive therapy for these diseases in calves, although several benefits have been found. When meloxicam was given at the onset of diarrhea, it led to improved appetite and body weight gain compared to control calves [120]. In addition, Phillip et al. [121] found that when meloxicam was given to diarrheic calves, improved general condition, feed intake, and fecal consistency was noted as well as a reduction in visceral pain compared to control calves. Flunixin meglumine has also been explored and, when given at the onset of diarrhea, those with blood in their stool had fewer days with diarrhea and antimicrobial treatments than untreated controls [122]. For respiratory disease, few trials have assessed the utility of providing an NSAID. Mahendran et al. [123] found that calves identified with a fever, used as a presumptive diagnosis for pneumonia, given flunixin meglumine had similar resolution and body weight gain to calves that received an antimicrobial. It is clear that there is a knowledge gap here to understand how painful these conditions are and how they need to be treated.

The prevention and treatment of disease on dairy farms represent areas of significant importance for dairy cattle quality assurance programs, as disease and illness on a farm impact many aspects of dairy production, from animal health and welfare, to biosecurity, food safety, and milk quality. Both the FARM program in the United States and the proAction program in Canada have numerous standards aimed at reducing the incidence and spread of disease on farms. In addition to Animal Care, each program also includes a pillar dedicated to Biosecurity and reducing disease incidence and transmission. The proAction program also includes a Traceability pillar, while the FARM Program has a specific pillar dedicated to Antibiotic Stewardship—both important aspects when considering disease. Both national programs require veterinary involvement and oversight of animal care protocols, management practices, and treatment of disease through a Herd Health Plan (HHP; FARM) and Cattle Health Declaration Form (proAction). The HHP and Cattle Health Declaration must include protocols specific to the treatment of common diseases and vaccination to prevent disease [10,11]. Both programs also require SOPs for pest control, a clean and well-ventilated calving area, and specific areas to segregate weak, sick, or injured animals in order to help prevent and reduce the spread of disease on a farm [10,11].

## 3. Where Do We Go from Here?

Throughout this review, it is apparent that despite overwhelming evidence that certain conditions are painful, such as disbudding and dehorning, there remains a need to increase the uptake of pain control. To better understand why pain management is not always implemented, there is a need to understand the perspectives of producers and veterinarians. In a survey of producers and veterinarians in Ireland, veterinarians gave lower pain scores than producers for conditions they saw more frequently, and the same was observed for conditions that producers saw more commonly compared to veterinarians [41]. This implies the potential for habituation decreasing the recognition of pain and potentially influencing the adoption of pain control for certain conditions where this effect is noted. In interviews with producers from Ontario, Canada about the use of pain control for disbudding and dehorning, it was identified that farmer attitudes surrounding the benefit of pain control, cost, and lack of education about procedures to provide pain control were primary barriers against providing pain control [123]. Lack of producer education was also found to be a barrier to the use of pain control for Brazilian farmers [124]. As veterinarians are highly regarded by producers to drive management changes [125] and alter the use of pain control [126], they should be engaged in the process to highlight the benefits of pain control and provide education to producers regarding proper methods for pain management.

Although cost is highlighted as a barrier to pain mitigation in Ontario dairy producers [127], in other parts of the world, it is likely that there is also a lack of options available for pain control [83]. Specifically, in the United States of America, only one NSAID, flunixin meglumine, is federally approved for pain mitigation [85]. Flunixin meglumine is approved as an anti-pyretic and anti-inflammatory, while transdermal flunixin is specifically approved for controlling fever associated with bovine respiratory disease and for pain relief in cases of foot rot in cattle [85]. However, it is not approved for pain relief in other common painful conditions or procedures discussed in this review, such as disbudding or castration. Similarly, regulations regarding the administration of anesthetics vary globally, with some regions restricting use to licensed veterinarians, while others allow trained farm personnel to administer local anesthetics under veterinary oversight. These regulatory differences further complicate pain management strategies, emphasizing the need for clear guidelines and education to ensure both compliance and effective pain relief. Action is needed to increase access to pain medications, expand approvals for pain relief options, and improve global pain management.

Concerns regarding drug withdrawal times and residues may also influence producer decisions on pain management strategies. Some producers, particularly those in organic dairy systems, have limited options for pain control due to certification restrictions. Additionally, uncertainty surrounding regulatory approvals for certain pain relief medications may further reduce uptake among conventional dairy producers. Addressing these concerns through education on withdrawal times, regulatory frameworks, and alternative pain management options may help increase the adoption of effective pain mitigation strategies.

A further challenge in the uptake of pain control is the inconsistency in pain assessment methods across studies. Disparities in pain scores may arise due to differences in behavioral and physiological indicators used to assess pain. Furthermore, variation in pain scoring systems complicates direct comparisons across studies. Emerging approaches, such as pharmacokinetic/pharmacodynamic modeling, offer promising methods to improve objectivity in pain assessment and optimize drug dosing strategies [128]. Hence, standardizing pain assessment tools across different conditions will be essential to better inform pain management strategies and improve uptake at the farm level.

Through this review, it also became clear that there are some conditions that require additional research to determine the best strategies to control pain. Specifically, metritis, diarrhea, respiratory disease, and castration need to be researched further to better understand how to control pain associated with these conditions or procedures.

## 4. Conclusions

Scientific literature highlights that there are many painful conditions and procedures that commonly occur in dairy cattle. These conditions and procedures require pain mitigation from an animal welfare standpoint and, in many cases, pain management carries additional advantages for the animal’s health and production. Pressure from consumers regarding animal welfare has provided further emphasis on the importance of reducing the incidence of pain in dairy cattle. Many dairy quality assurance programs have implemented requirements for pain control for procedures like castration, dehorning, and disbudding; however, there is still room for elevated expectations around best practices for managing pain in dairy cattle. Through this review, scientific evidence for the use of pain mitigation has been laid out in several painful conditions, including common procedures and disease. Further, the current uptake of pain management among producers and veterinarians is described. Although, in some conditions and procedures, it is clear that there are advantages to providing pain control, the actual uptake lags behind, meaning there is still a need to communicate the benefits of pain control for these conditions to producers and veterinarians. For other conditions and procedures, more evidence is needed to determine the best methods for pain management. Overall, despite how common painful conditions and procedures occur in dairy cattle, pain itself does not need to be nearly as common, and if dairy production wishes to maintain a good reputation in the eyes of consumers, it should not be.

## Figures and Tables

**Table 1:** Common painful conditions and management strategies to mitigate pain in dairy cattle.

Painful Condition	Signs of Pain	Pain Alleviation Methods
**Disbudding/Dehorning**	Increased heart rate and cortisol, reduced feed intake [13,14]	Local anesthetic, NSAIDs, breeding polled cattle [15,16,17,18]
**Castration**	Kicking, increased cortisol, reduced weight gain, abnormal posture [19,20,21]	Local anesthetic, NSAIDs, multimodal analgesia [22,23,24,25,26]
**Lameness**	Reluctance to move, weight shifting, arched back, abnormal gait, increased heart rate and cortisol [27,28,29]	Corrective trimming, hoof blocks, NSAIDs, gait scoring and early intervention [30,31,32,33,34,35,36,37,38,39]
**Parturition/Dystocia**	Increased lying down to standing transitions, decreased feed intake, less self-grooming [40,41,42]	NSAIDs (ketoprofen, meloxicam), early and appropriate intervention [43,44,45,46,47,48,49,50,51]
**Abdominal Surgeries (e.g., C-section, LDA surgery)**	Reduced feed intake, less lying time, more tail flicking, lower heart variability [46,52,53,54]	Local anesthetic, NSAIDs (meloxicam, ketoprofen) [46,52,55]
**Mastitis**	Increased sensitivity, elevated heart and respiratory rate, higher cortisol [56,57,58]	NSAIDs (meloxicam, flunixin meglumine), supportive therapy [58,59,60,61,62,63,64,65,66]
**Metritis**	Arched back, alteration in heart rate [67,68,69,70,71,72,73]	NSAIDs (meloxicam, flunixin meglumine), supportive therapy [68,69,70,71,72,73]
**Calf Diarrhea**	Lethargy, reduced milk intake, prolonged lying [74,75]	NSAIDs (meloxicam, flunixin meglumine), supportive therapy [76,77,78]
**Respiratory Disease**	Lethargy, reduced milk intake, prolonged lying [74,75]	NSAIDs, supportive care [79]

Note: The pain management strategies listed in this table include both approved and extra-label uses of analgesics and anesthetics. Regulations regarding the administration of local anesthetics (e.g., lidocaine) vary by country, with some regions restricting use to licensed veterinarians, while others permit administration by trained farm personnel under veterinary oversight. Additionally, regulatory approvals for pain relief medications differ globally. Producers and veterinarians should consult regional regulatory authorities (e.g., FDA, EMA, CFIA) to confirm approved indications and legal administration requirements before use.

**Table 2:** Adoption of pain management strategies for painful conditions in dairy cattle.

Painful Condition	Local Anesthesia-Veterinarians (%)	Local Anesthesia-Producers (%)	NSAIDs/Systemic Analgesia-Veterinarians (%)	NSAIDs/Systemic Analgesia-Producers (%)	References
**Disbudding/Dehorning**	65–98%	21–62%	39–56%	24–35%	[80,81,82,83,84,85,86]
**Castration**	32–48%	10–13%	25–42%	8–10%	[9,85]
**Lameness**	N/A	N/A	60–75%	80%	[9,85]
**Parturition/Dystocia**	N/A	N/A	60–68%	73%	[9,85]
**Abdominal Surgeries**	89%	N/A	61–77%	61–98%	[9,85,87]
**Mastitis**	N/A	N/A	13–42%	26%	[85,87]

Note: N/A indicates that no estimates are available.
